# Supplementary material for: Chiral superstructures of inorganic nanorods by macroscopic mechanical grinding
Source: Nat Commun. 2022 Oct 4;13:5844. doi: 10.1038/s41467-022-33638-6 (PMC9532428; doi:10.1038/s41467-022-33638-6)
Supplement: Supplementary file 1 — Supplementary Information [file 41467_2022_33638_MOESM1_ESM.pdf]

Supplementary Information for

Chiral superstructures of inorganic nanorods by macroscopic mechanical grinding

Zhiwei Yang[1,2], Yanze Wei[1,2], Jingjing Wei[1], Zhijie Yang[1*]

[1]Key Laboratory of Colloid and Interface Chemistry, Ministry of Education, School of Chemistry and Chemical Engineering, Shandong University, Jinan 250100, P.R. China.

[2]These authors contributed equally: Zhiwei Yang, Yanze Wei

*Corresponding author: zyangchem@sdu.edu.cn

Table of contents

1. Supplementary Figures1-24……………………………………………S3

2. Supplementary Table 1 ………………………………………………..S27

3. Supplementary Notes1-5………………………………………………S28

4. Supplementary References……………………………………………..S34

Supplementary Figures

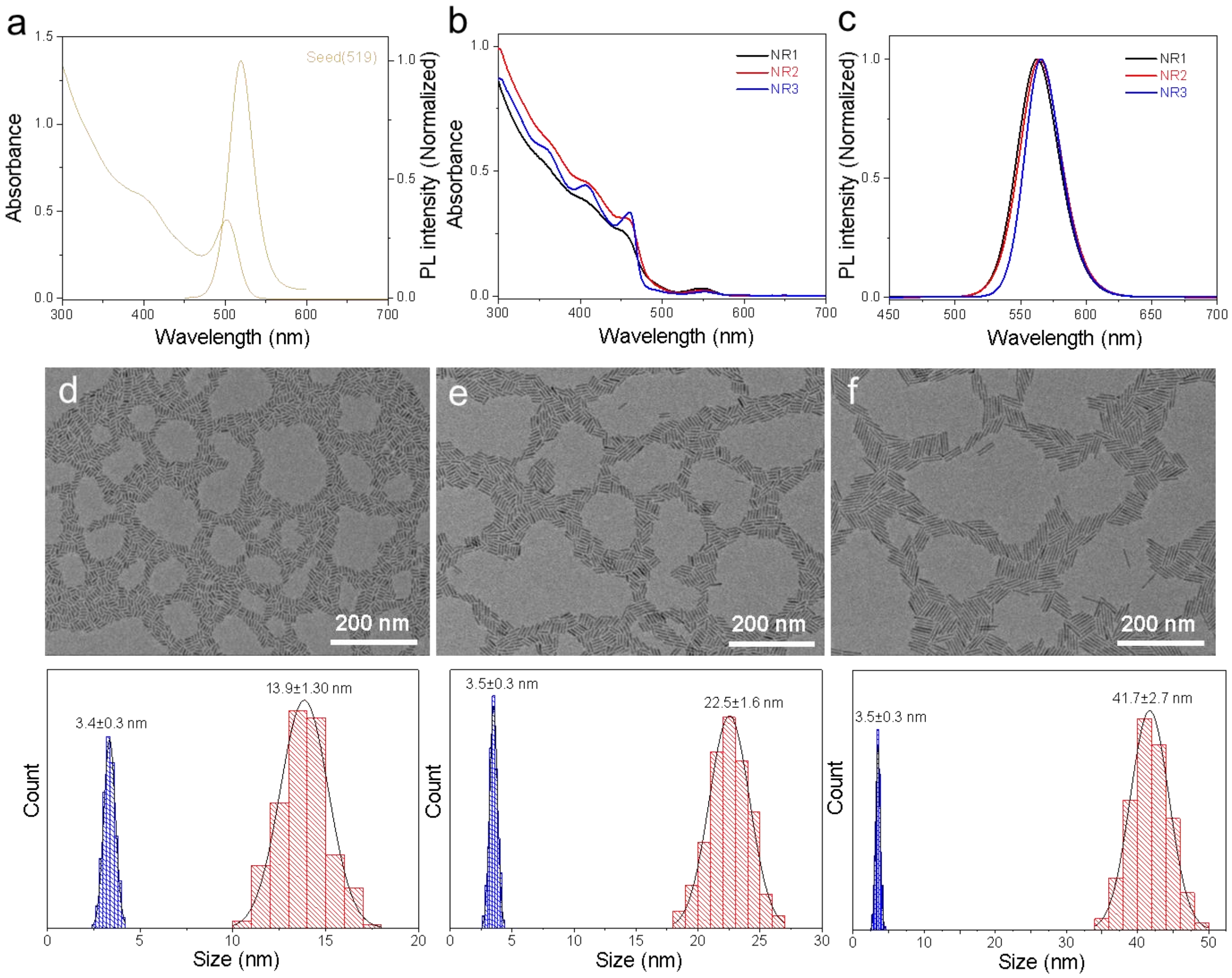

Supplementary Fig. 1. Spectral and morphological characterization of CdSe-CdS NRs with different lengths. The UV-vis absorption spectra and fluorescence spectra of seed(519) (a). The UV-vis absorption spectra (b) and fluorescence spectra (c) of NR1, NR2 and NR3. TEM images and size histograms of NR1 (d), NR2 (e) and NR3 (f), respectively. (The width and length of NRs were determined by counting over 200 particles for respective NR1, NR2 and NR3.)

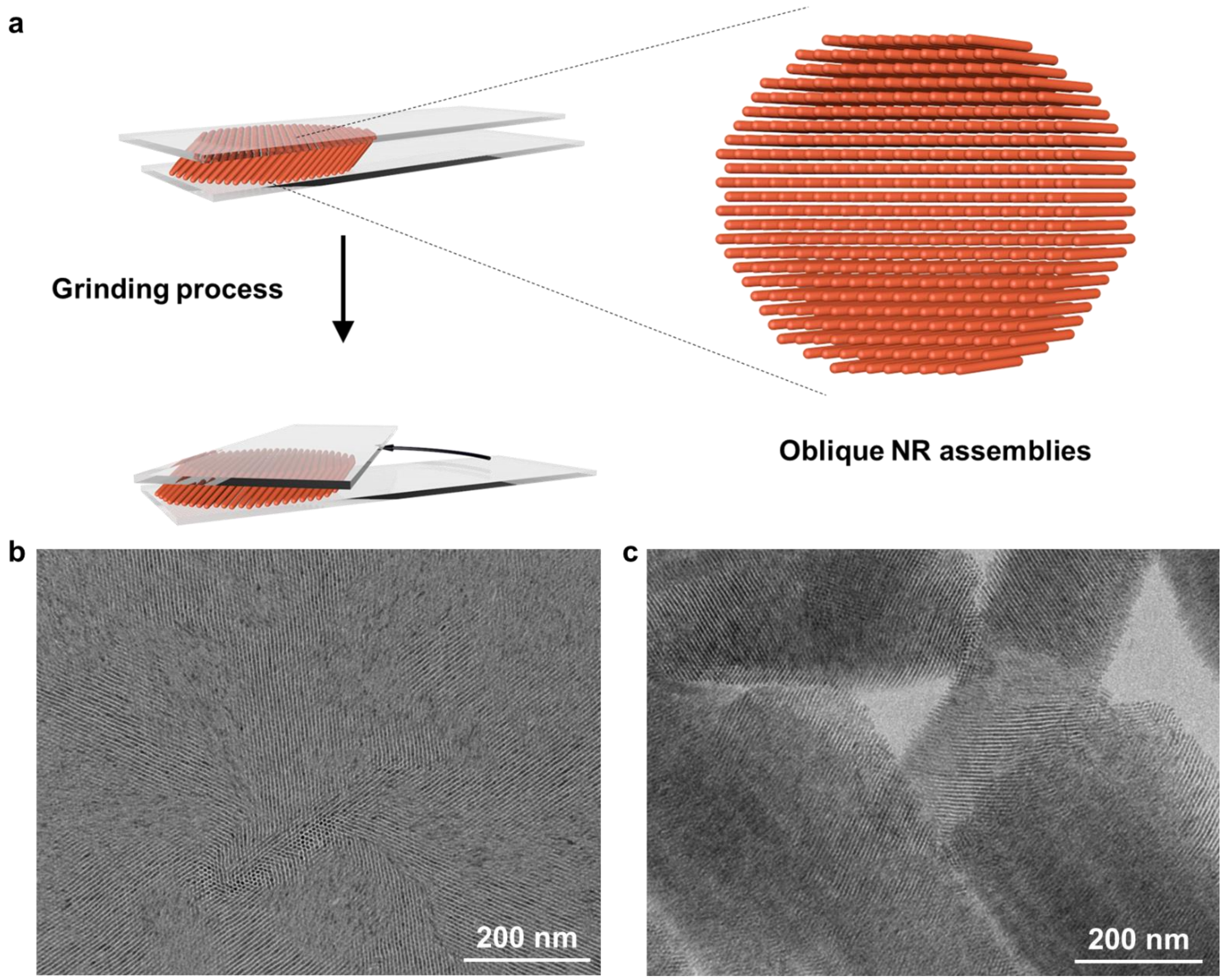

Supplementary Fig. 2. Assembly morphology of NR2 and NR3. (a) Scheme for the grinding process of the NR2 or NR3 assemblies, which showed the oblique assembling behavior. TEM images of the NR2 (b) and NR3 (c) assemblies used for the grinding experiments.

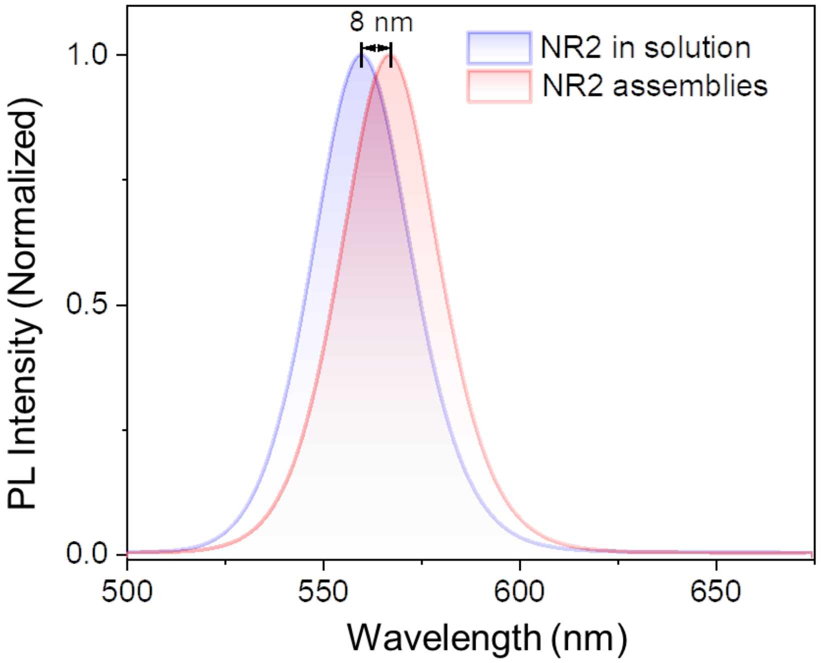

Supplementary Fig. 3. PL spectra of NR2 in solution and in assembled state.

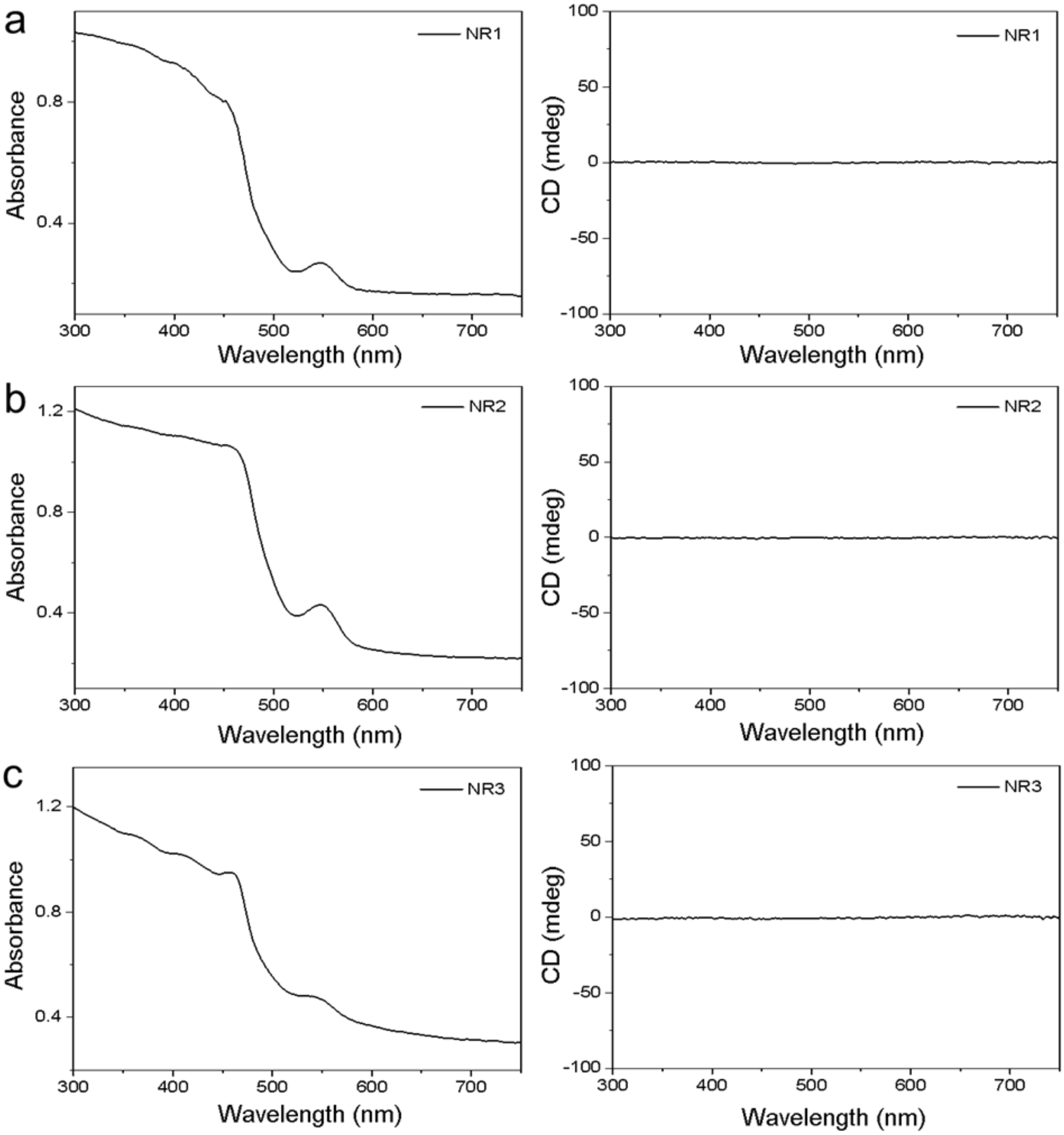

Supplementary Fig. 4. Chirality Analysis of the CdSe-CdS NRs with different Length. The CD spectra and UV-vis absorption spectra of NR1 (a), NR2 (b), and NR3 (c) assemblies without mechanical grinding.

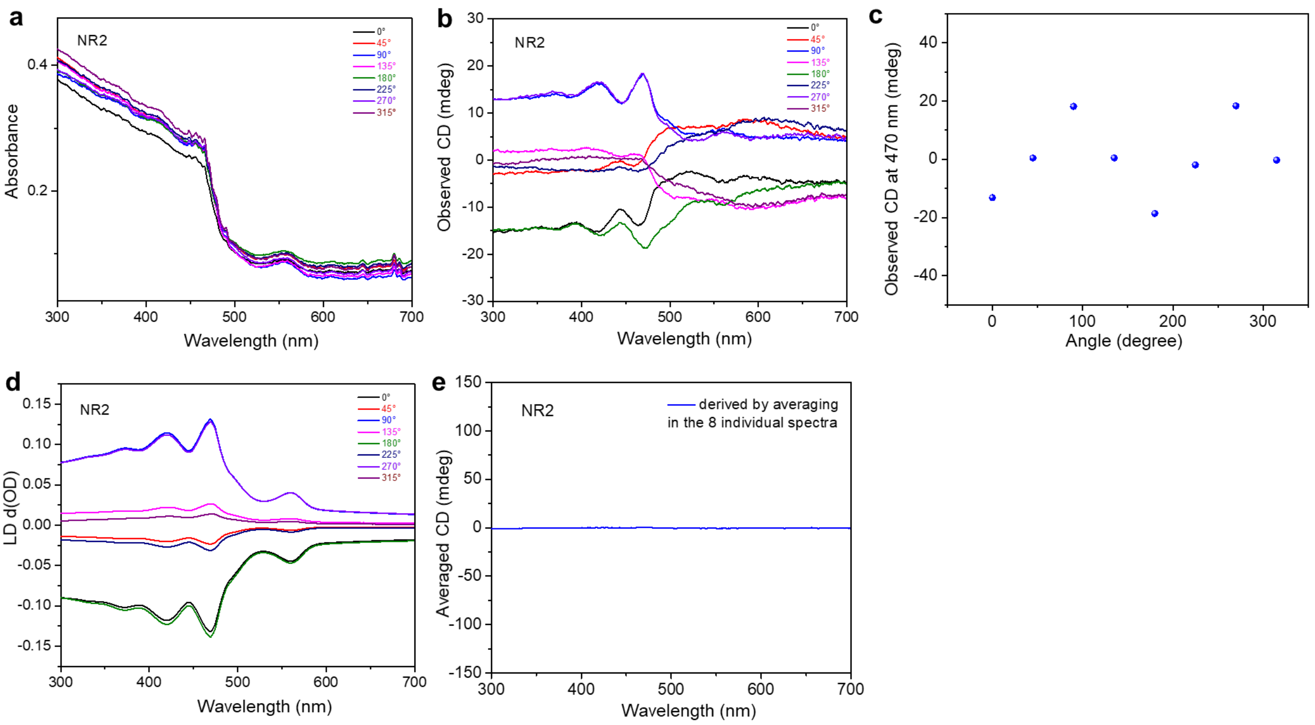

Supplementary Fig. 5. Rubbing the NR2 assemblies in a linear fashion (back and forth): The UV−vis absorption spectra (a), observed CD spectra (b), the rotating angle dependent observed CD at 470 nm (c), and LD spectra (d) of NR2 at various angles (recording every 45° from 0 to 315°). (e) The averaged CD spectra from averaging the eight individual spectra in (b).

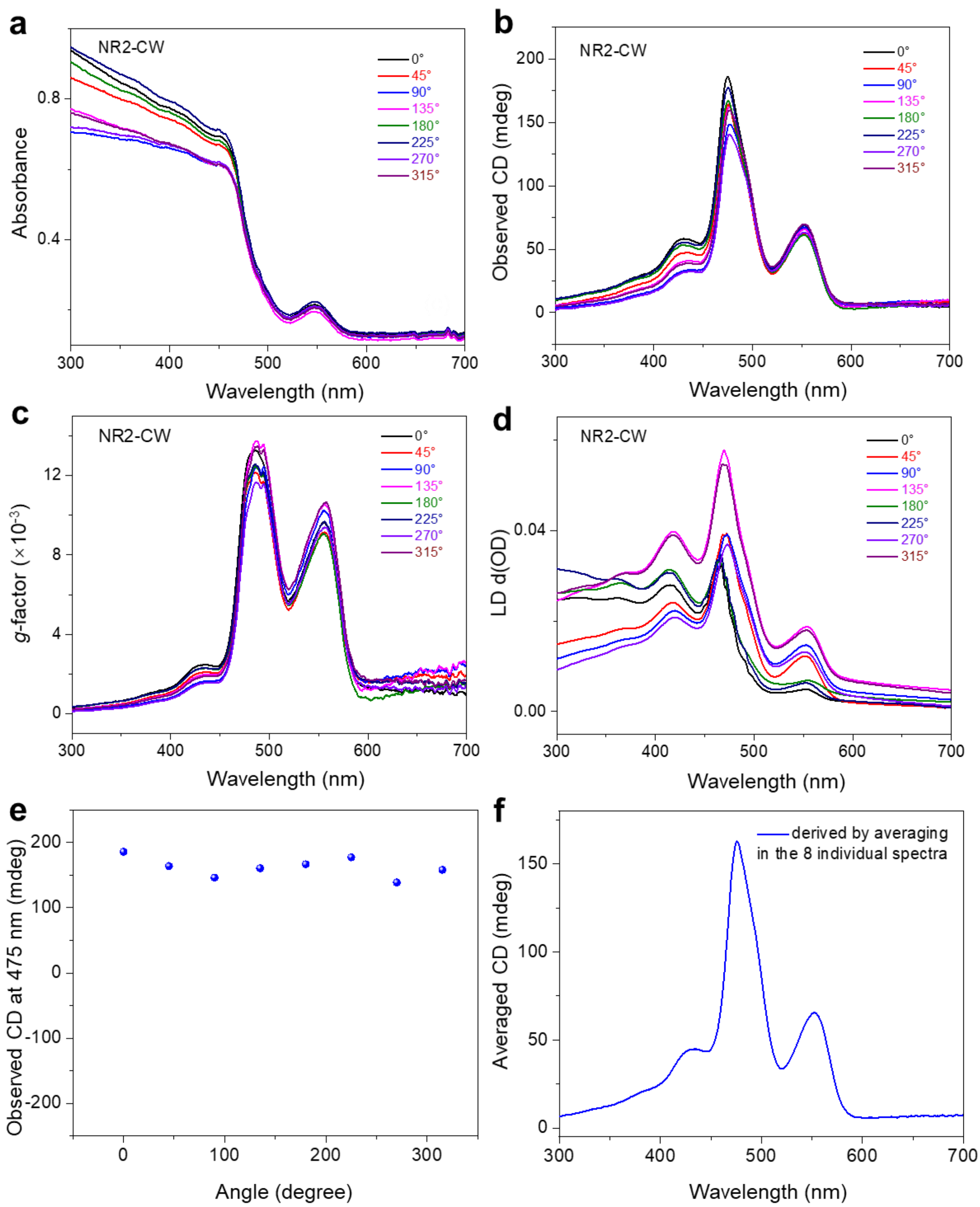

Supplementary Fig. 6. CD and LD spectra for NR2 assemblies after mechanical grinding: UV−vis absorption spectra (a), CD spectra (b), g-factor curves (c) and LD spectra (d) at various rotating angles (recording every 45° from 0 to 315°). (e) The rotating angle dependent observed CD at 475 nm. (f) The averaged CD spectrum from averaging the eight individual spectra in (b).

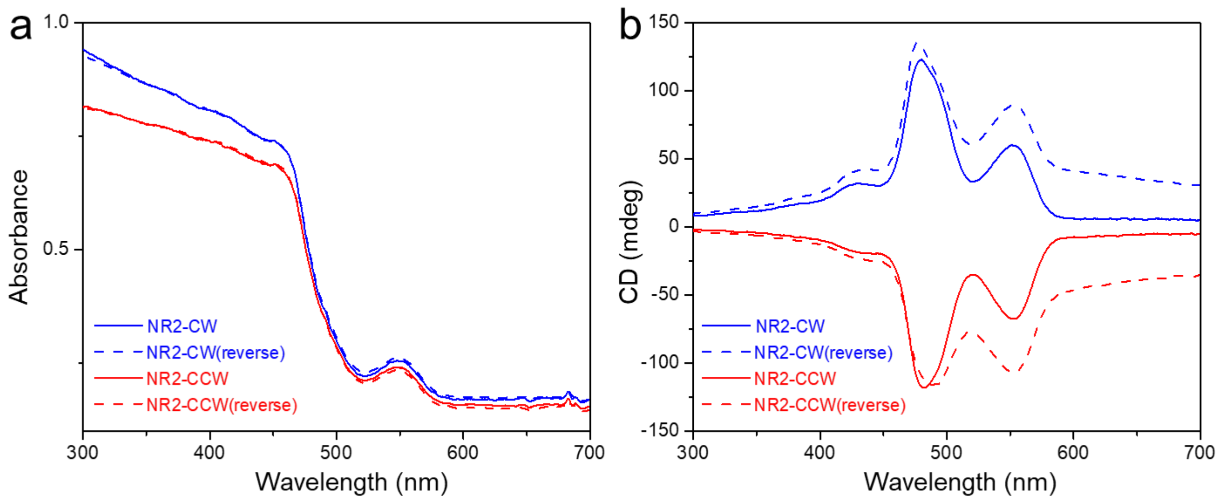

Supplementary Fig. 7. UV-vis spectra (a) and CD spectra (b) of NR2(CW) and NR2(CCW) samples measured at two opposites faces of the quartz substrate.

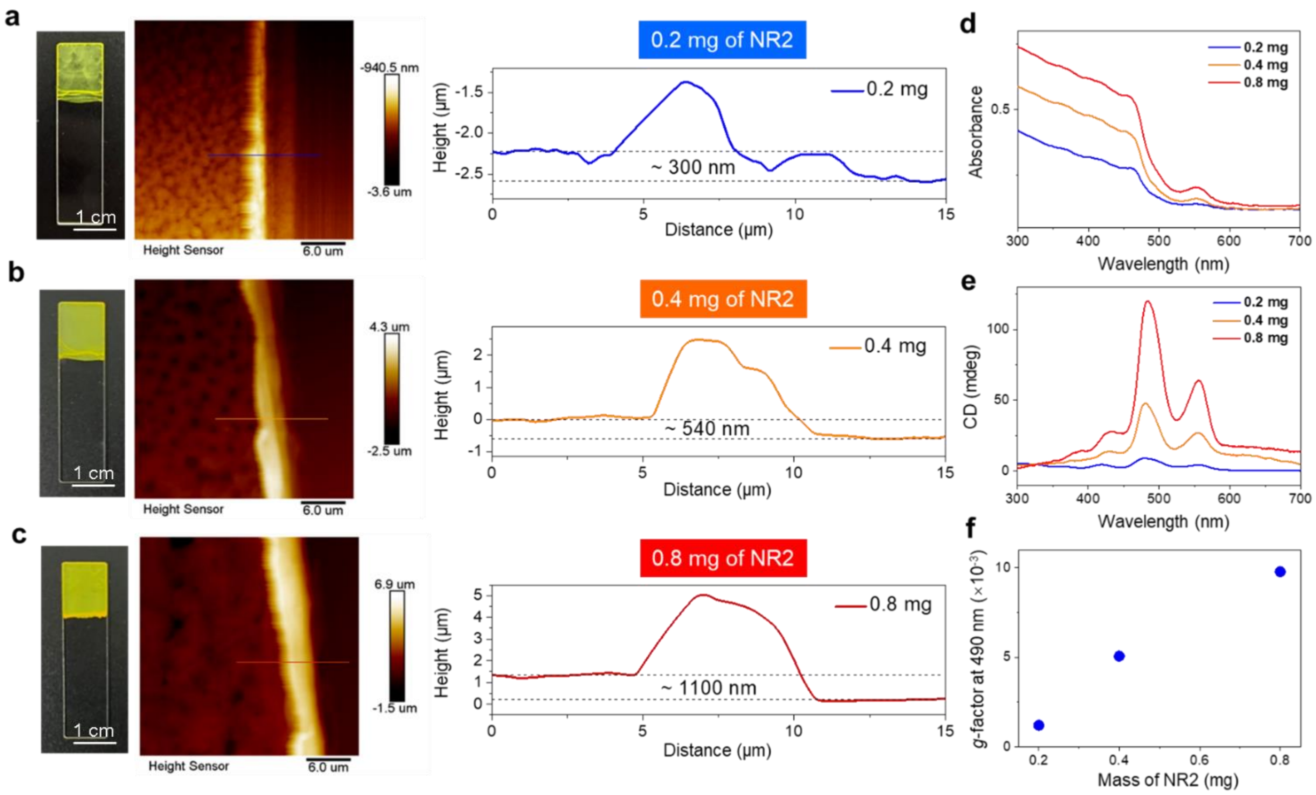

Supplementary Fig. 8. Effects of CdSe-CdS NRs with different thicknesses on chiral response. Optical image (left), AFM image (middle), and the corresponding height profile (right) of NR assemblies of various thicknesses (a) ~300 nm from deposition of 0.2 mg of NR2; (b) ~540 nm from deposition of 0.4 mg of NR2; (a) ~1100 nm from deposition of 0.8 mg of NR2. The absorption (d) and CD (e) spectra of the NR2 assemblies differing by the film thicknesses (mass of NR2) after grinding processing. (f) The plot of *g*-factor at 490 nm versus the mass of NR2.

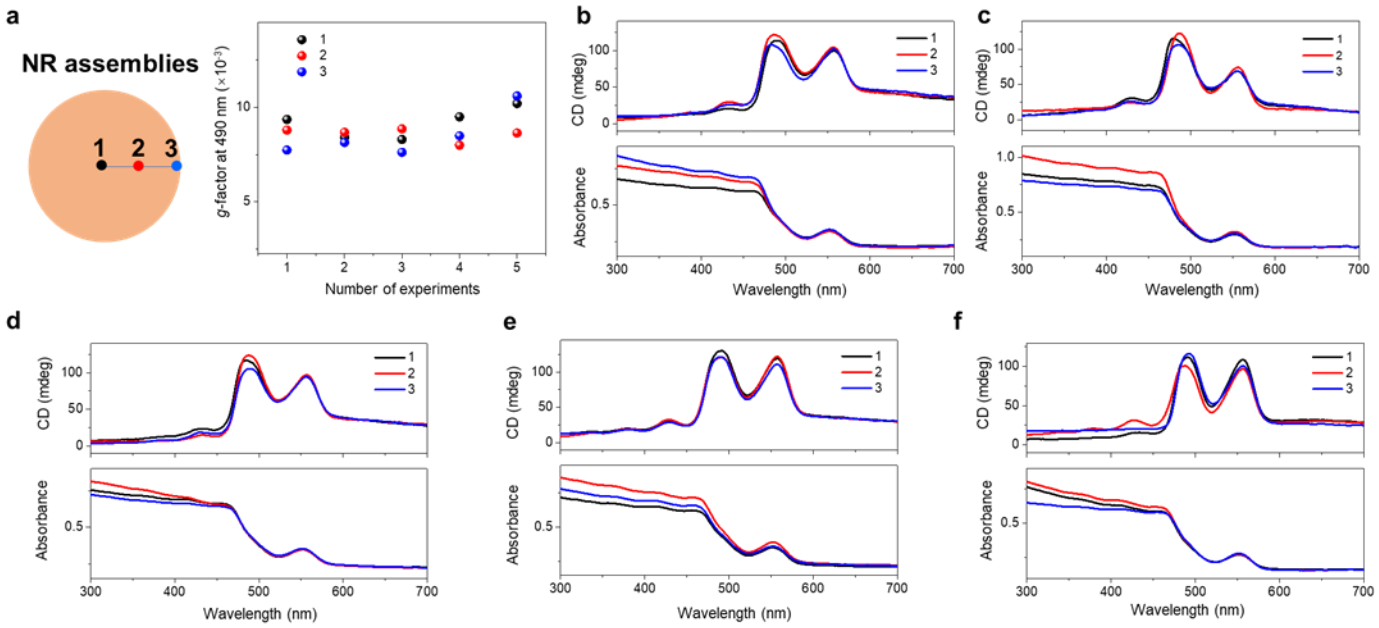

Supplementary Fig. 9. CD signal at different positions of the sample. (a) the scheme for the various regions of the NR assemblies for the CD measurements (left) and the g-factor at 490 nm of three different regions from five independent experiments (right); (b-f) CD and absorbance spectra of NR assemblies after grinding processing from five independent experiments.

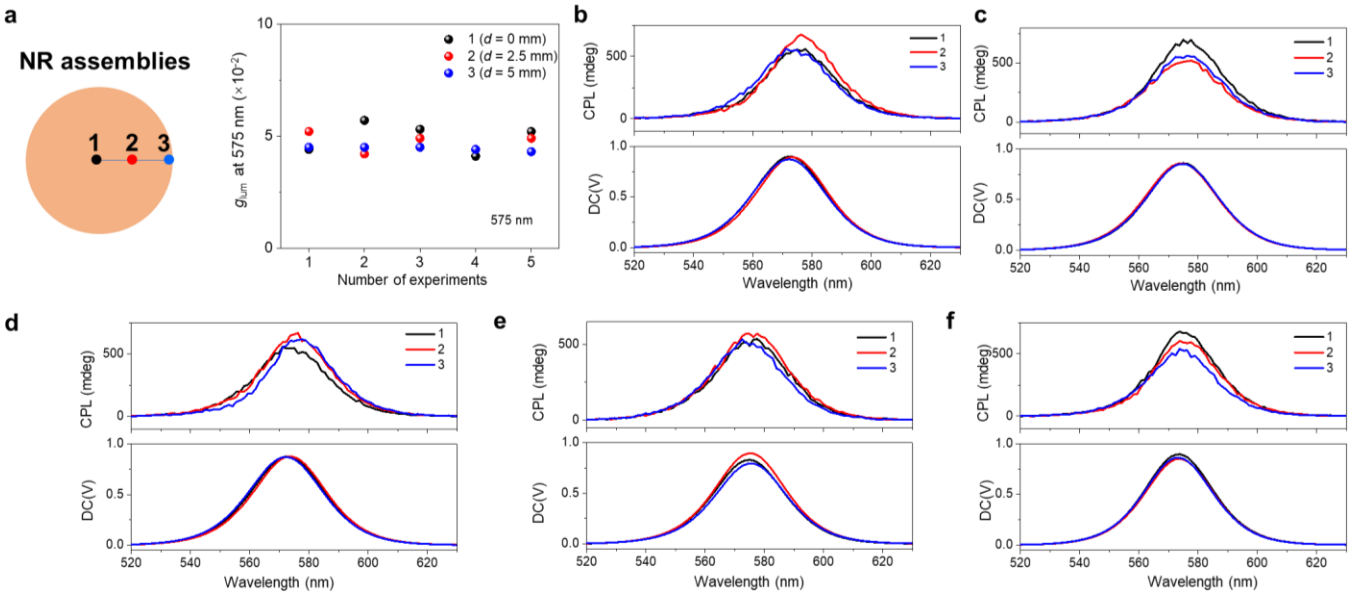

Supplementary Fig. 10. CPL signal at different positions of the sample. (a) the scheme for the various regions of the NR assemblies for the CPL measurements (left) and the g_{lum} at 575 nm of three different regions from five independent experiments (right); (b-f) CPL and PL spectra of NR assemblies after grinding processing from five independent experiments.

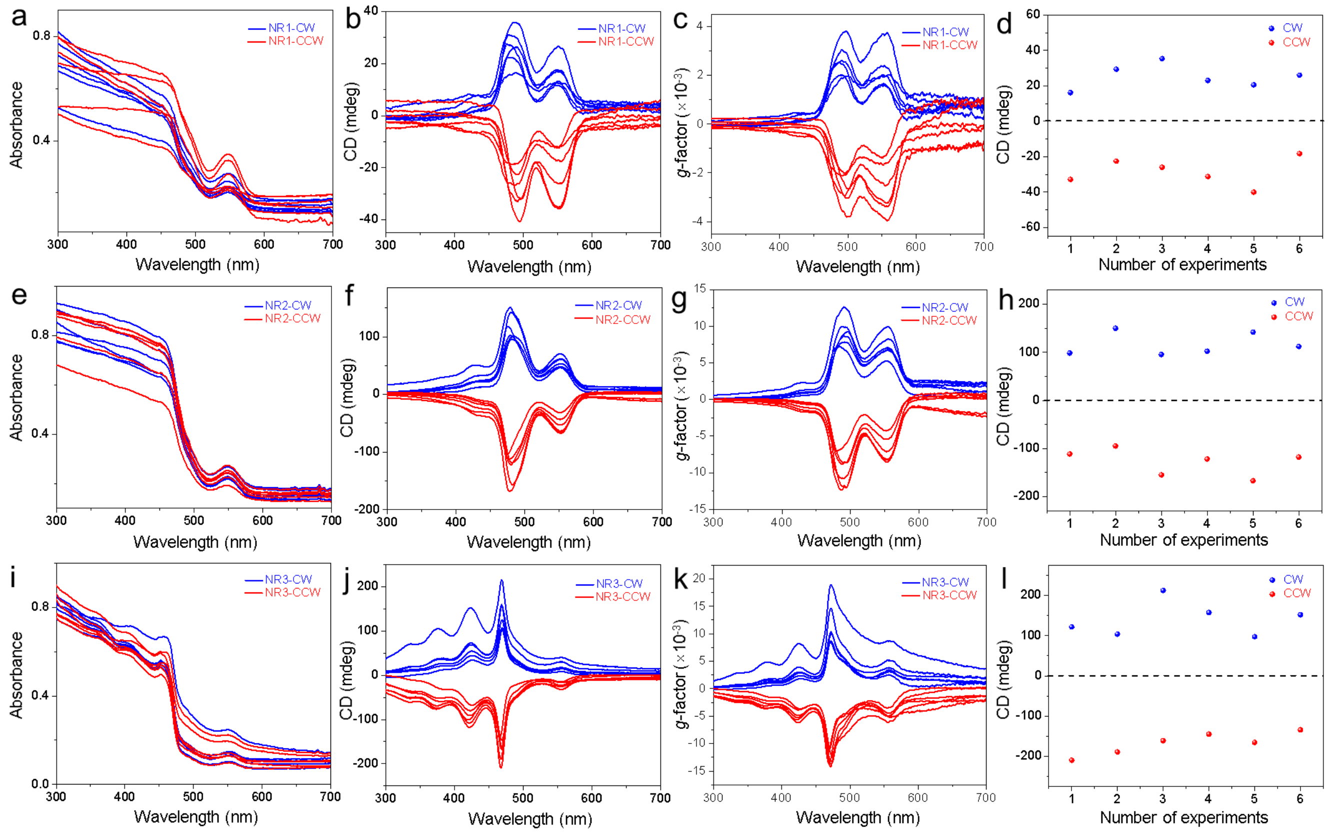

Supplementary Fig. 11. Statistical of chiral signals after mechanical grinding of CdSe-CdS NRs with different lengths. Repeated experiments of NR assemblies from NRs differing by the lengths: (a, b, c) UV-vis absorption spectra, CD spectra and *g*-factor of NR1 and (d) Statistics of CD values at 490 nm for NR1. (e, f, g) UV-vis absorption spectra, CD spectra and *g*-factor of NR2 and (h) Statistics of CD values at 490 nm for NR2. (i, j, k) UV-vis absorption spectra, CD spectra and *g*-factor of NR3 and (l) Statistics of CD values at 490 nm for NR3.

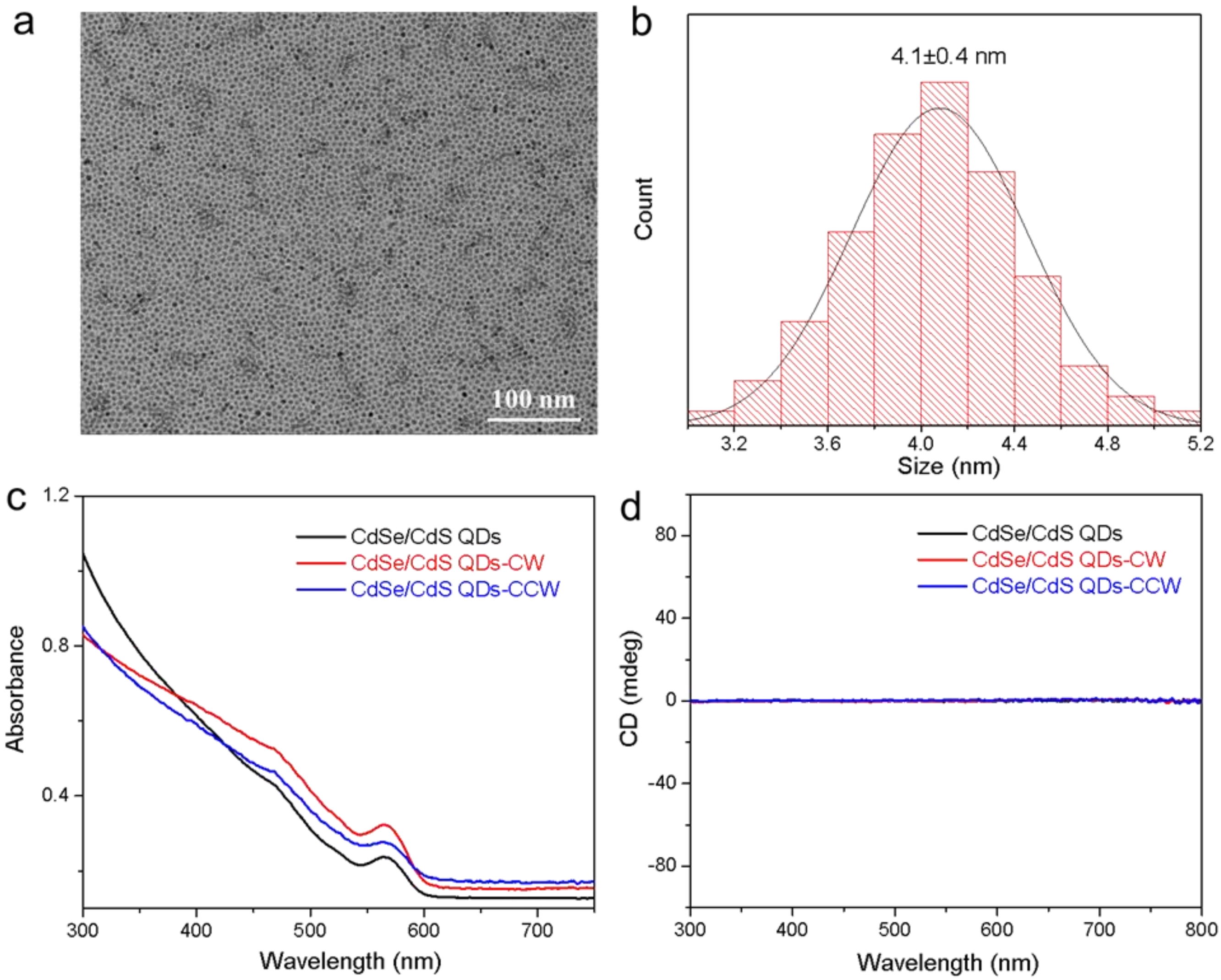

Supplementary Fig. 12. Morphology and spectroscopic determination of CdSe-CdS QDs. CdSe/CdS QDs assemblies subjected to the mechanical grinding. (a, b) TEM images and size histograms of CdSe/CdS QDs. (c, d) UV−vis absorption spectra and CD spectra of CdSe/CdS QDs, CdSe/CdS QDs-CW and CdSe/CdS QDs-CCW.

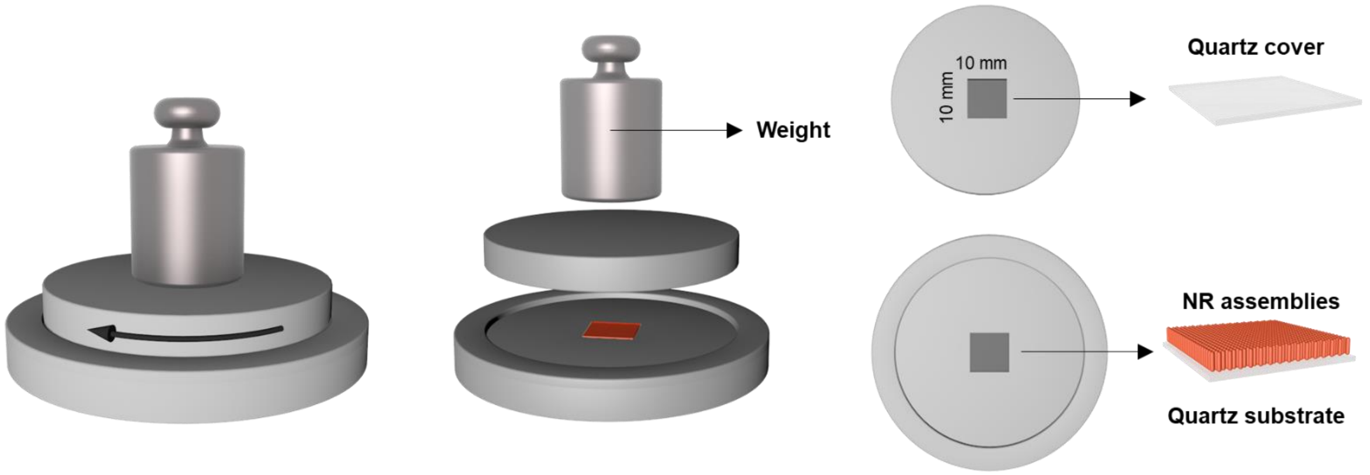

Supplementary Fig. 13. Scheme for the homemade device for controlling the applied pressure during the grinding processing of NR assemblies. The material of the device was made of Teflon. The size of the quartz substrate was 10×10 mm^2.

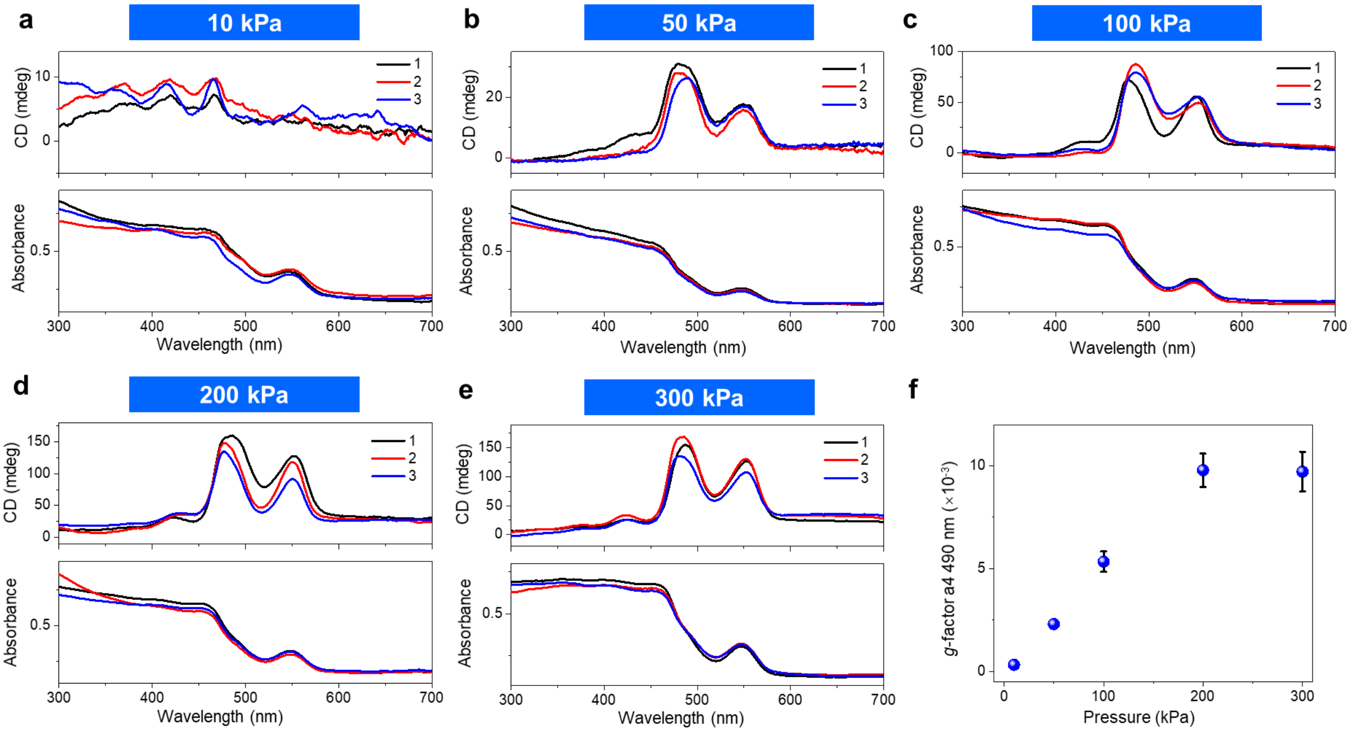

Supplementary Fig. 14. The effect of different pressures on the chiral signal of NR2. The grinding processing of NR assemblies under various pressure: (a) 10 kPa; (b) 50 Pa; (c) 100 kPa; (d) 200 kPa; (e) 300 kPa. (f) The plot of *g*-factor at 490 nm versus the applied pressure. Error bars in (f) are determined by standard errors (SE).

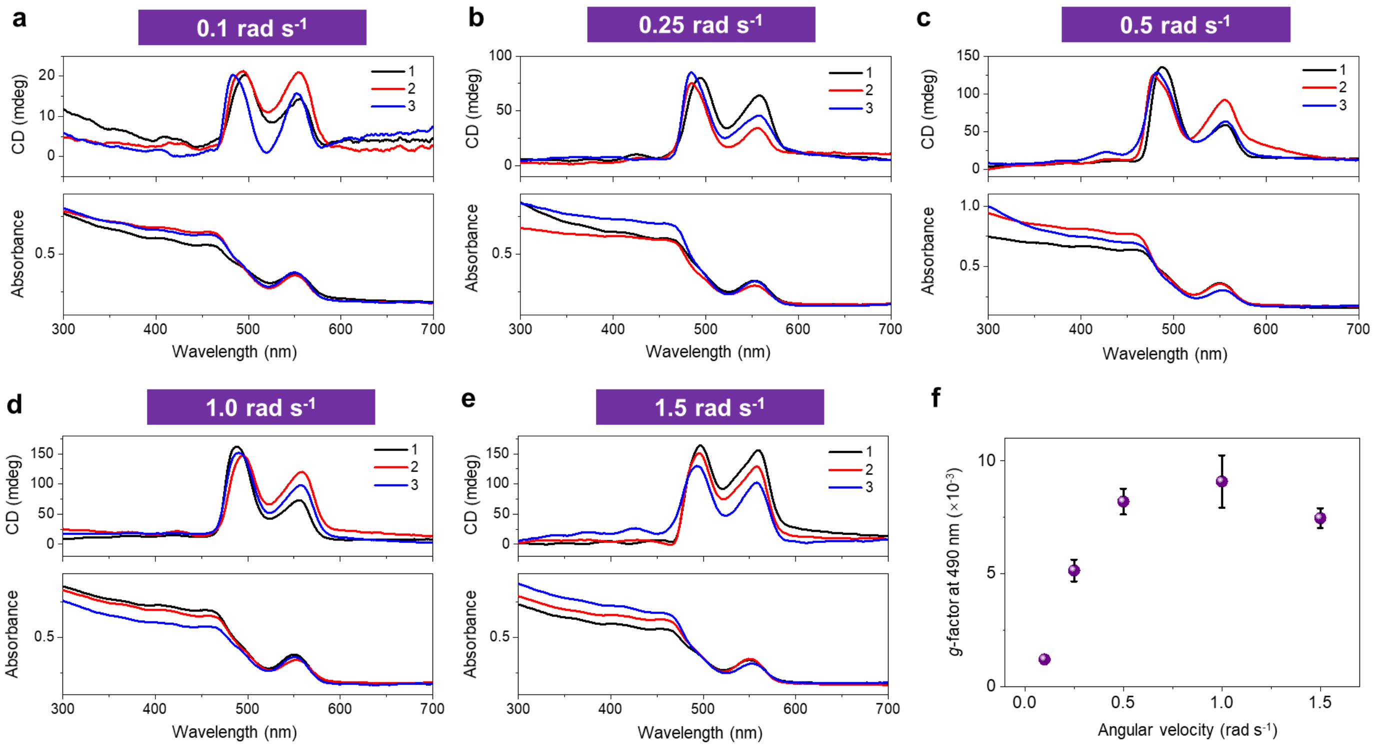

Supplementary Fig. 15. The effect of different angular velocity on the chiral signal of NR2. Control experiments carried out at various rotating speed: (a) 0.1 rad s^{-1}; (b) 0.25 rad s^{-1}; (c) 0.5 rad s^{-1}; (d) 1.0 rad s^{-1}; (e) 1.5 rad s^{-1}; (f) The plot of *g*-factor at 490 nm versus the angular velocity. Error bars in (f) are determined by standard errors (SE).

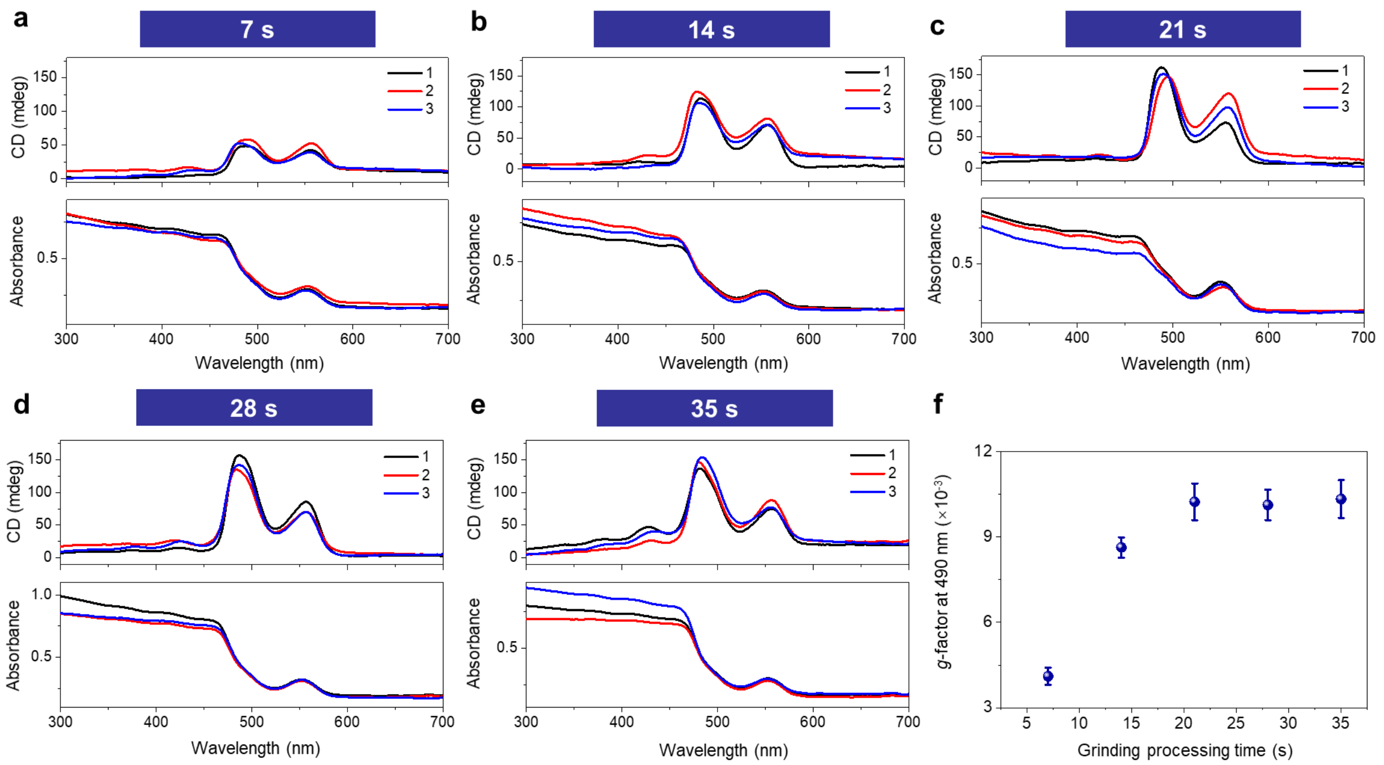

Supplementary Fig. 16. The effect of different grinding times on the chiral signal of NR2. Grinding of NR2 assemblies at various processing times: (a) 7 s; (b) 14 s; (c) 21 s; (d) 28 s; (e) 35 s. (f) The plot of *g*-factor at 490 nm versus the processing time. Error bars in (f) are determined by standard errors (SE).

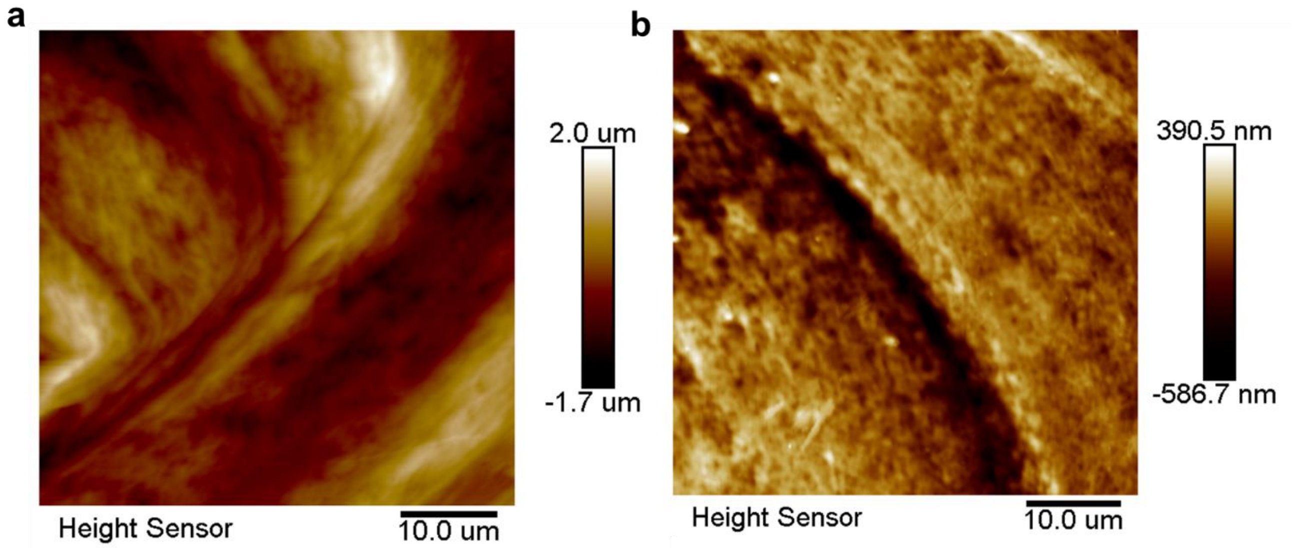

Supplementary Fig. 17. Additional AFM images of the chiral NR2 assemblies after the CW grinding.

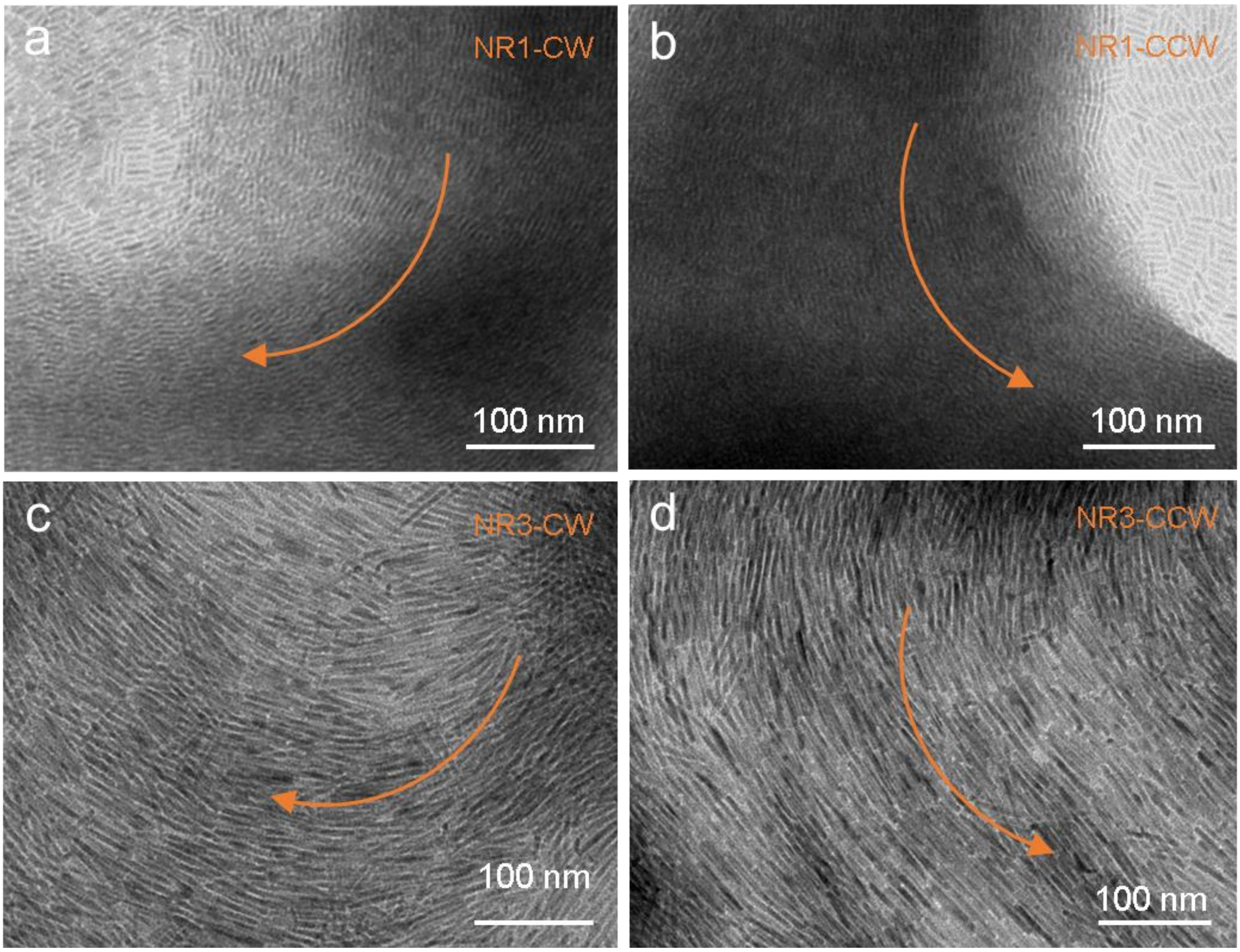

Supplementary Fig. 18. TEM images of (a) NR1-CW and (b) NR1-CCW, (c) NR3-CW and (d) NR3-CCW.

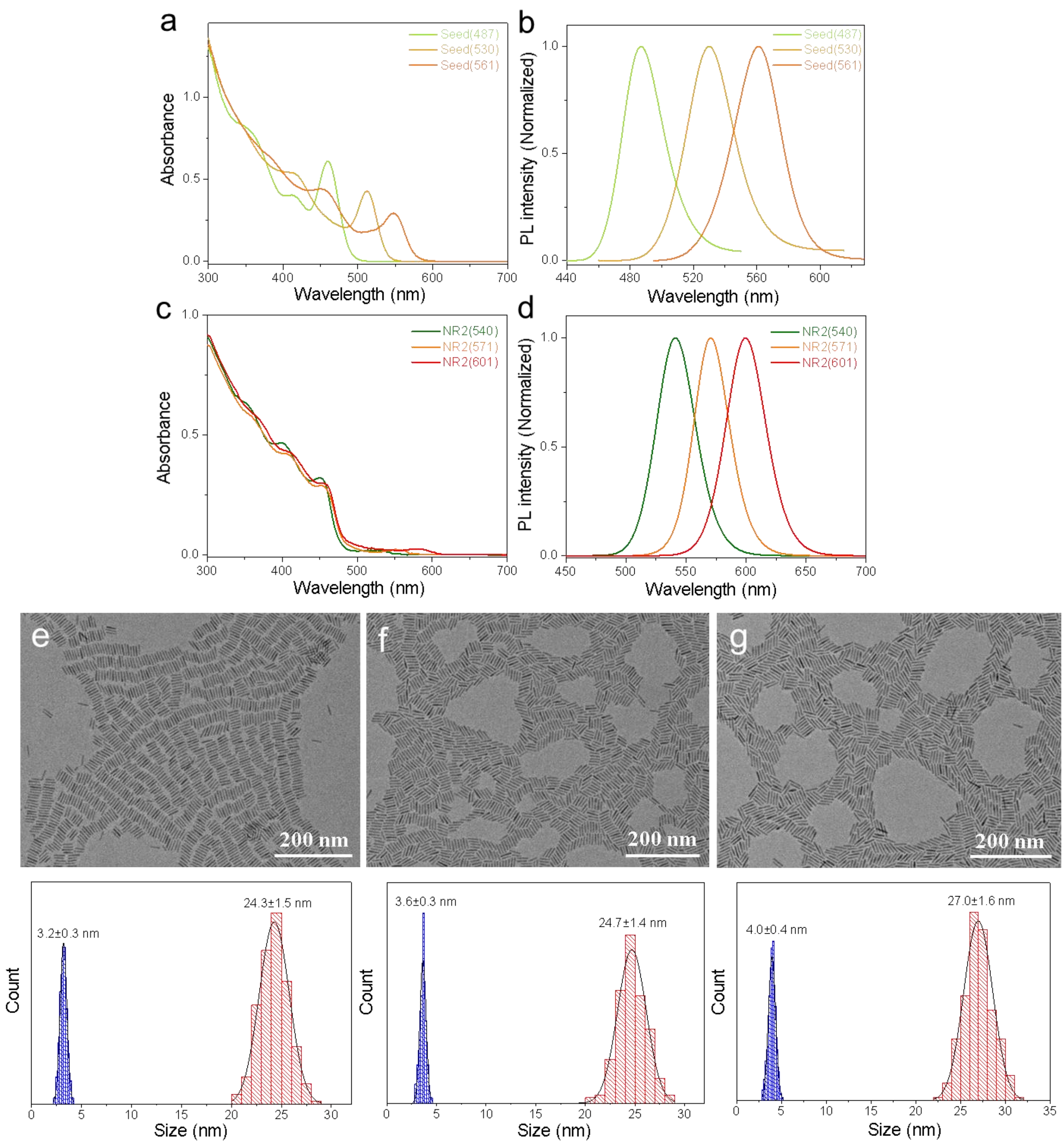

Supplementary Fig. 19. Spectral and morphological characterization of CdSe-CdS NRs with different fluorescence emission. UV-vis absorption spectra (a) and fluorescence spectra (b) of seed(487), seed(530) and seed(561). UV-vis absorption spectra (c) and fluorescence spectra (d) of NR2(540), NR2(571) and NR2(601). TEM images and size histograms of (e) NR2(540), (f) NR2(571) and (g) NR2(601), respectively. (The width and length of NRs were determined by counting over 200 particles for respective NR2(540), NR2(571) and NR2(601).)

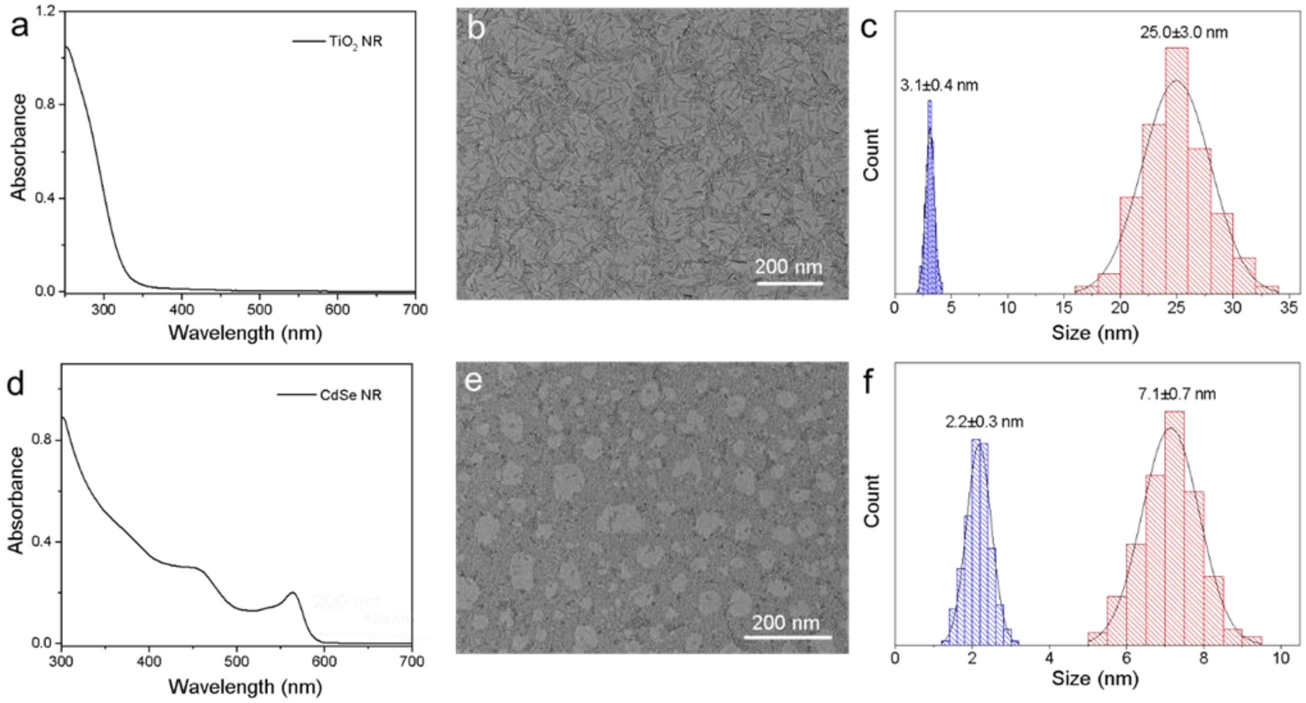

Supplementary Fig. 20. Spectral and morphological analysis of different nanorods. The UV-vis absorption spectra, TEM images and size histograms of TiO_2 NRs (a, b, c) and CdSe NRs (d, e, f) (The width and length of TiO_2 NRs and CdSe NRs were determined by counting over 200 particles).

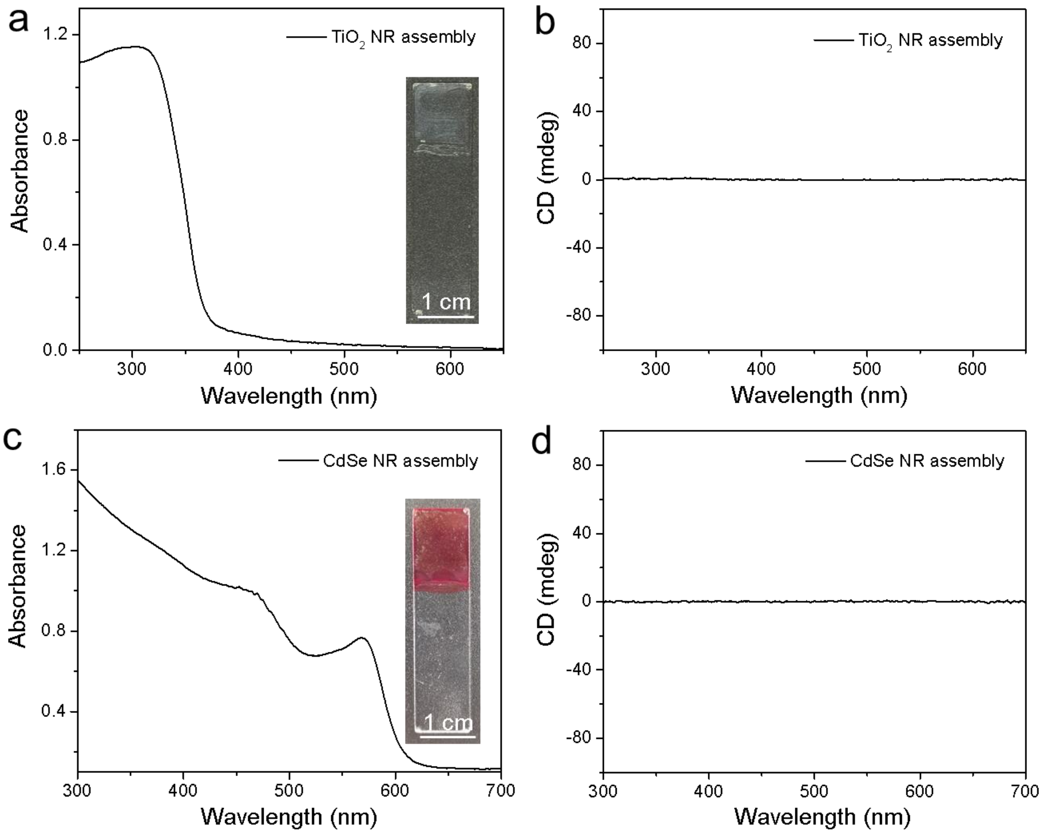

Supplementary Fig. 21. The UV-vis absorption spectra and CD spectra of TiO_2 NRs (a, b) and CdSe NRs (c, d) assemblies without mechanical grinding treatments.

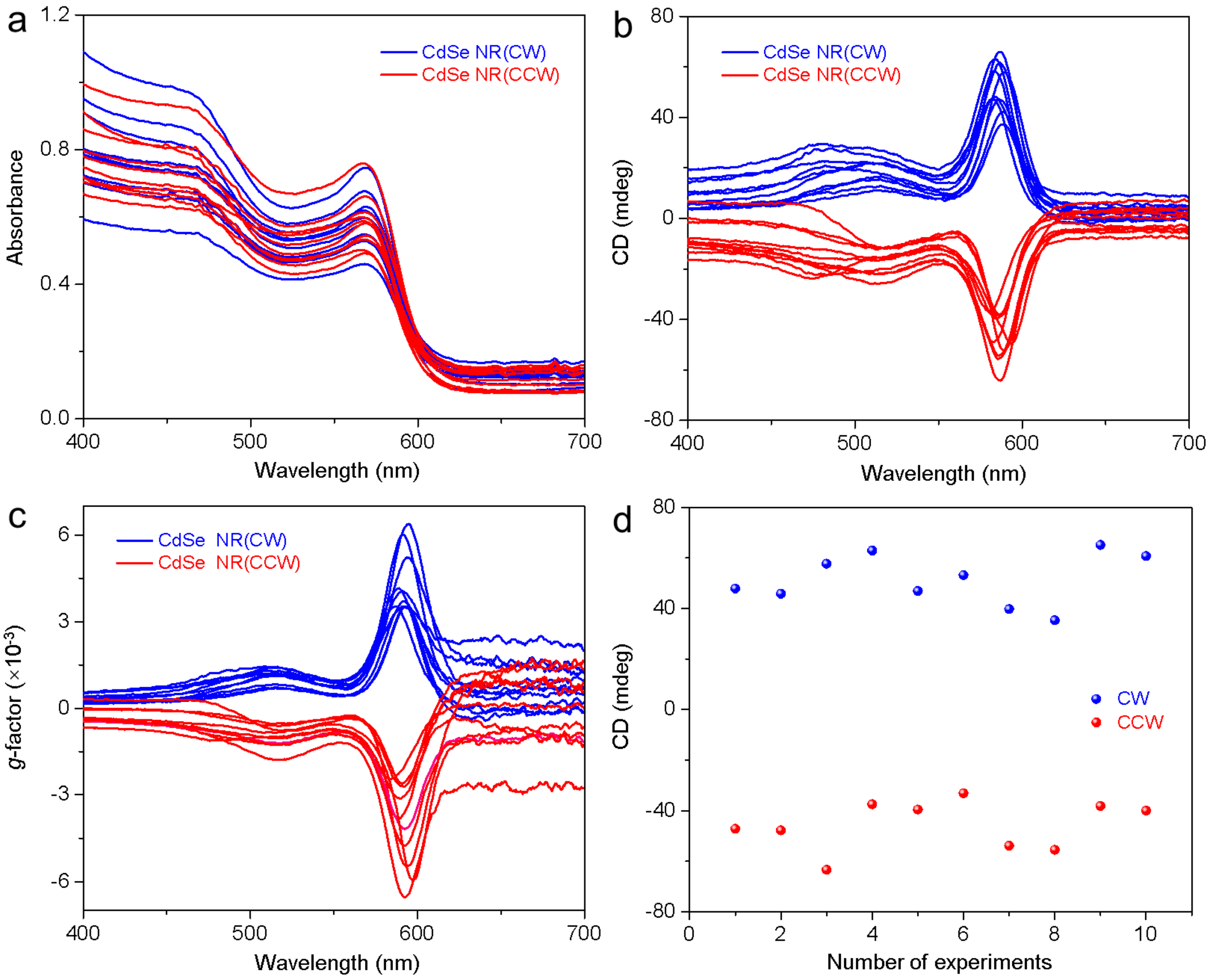

Supplementary Fig. 22. Repeated mechanical grinding experiments for CdSe NR assemblies: (a) UV-vis absorption spectra, (b) CD spectra and (c) *g*-factor curves of CdSe NRs (CW) and CdSe NRs (CCW). (d) CD values at 585 nm for CdSe NR (CW) and CdSe NR (CCW), respectively.

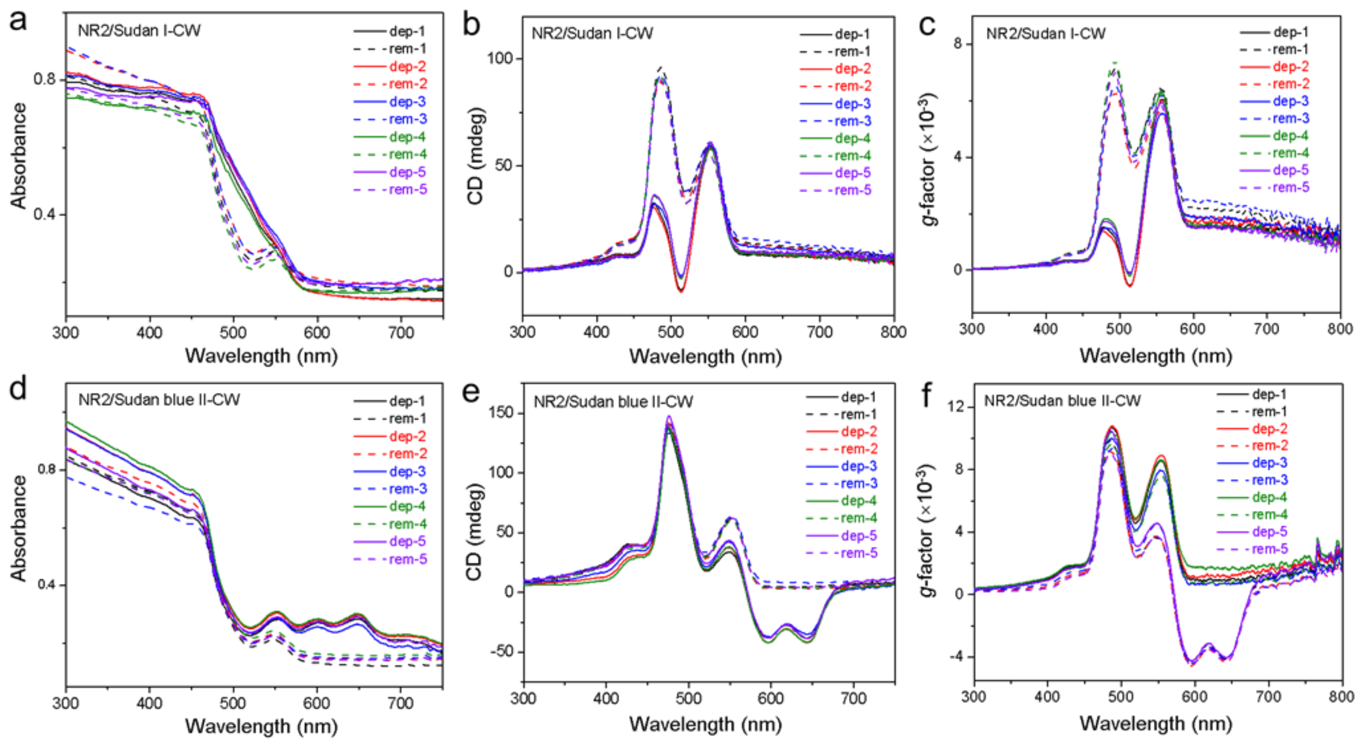

Supplementary Fig. 23. Dye deposition-removal experiments: UV-vis absorption spectra, CD spectra and *g*-factor curves of NR2/Sudan I-CW (a, b, c) and NR2/Sudan blue II-CW (d, e, f).

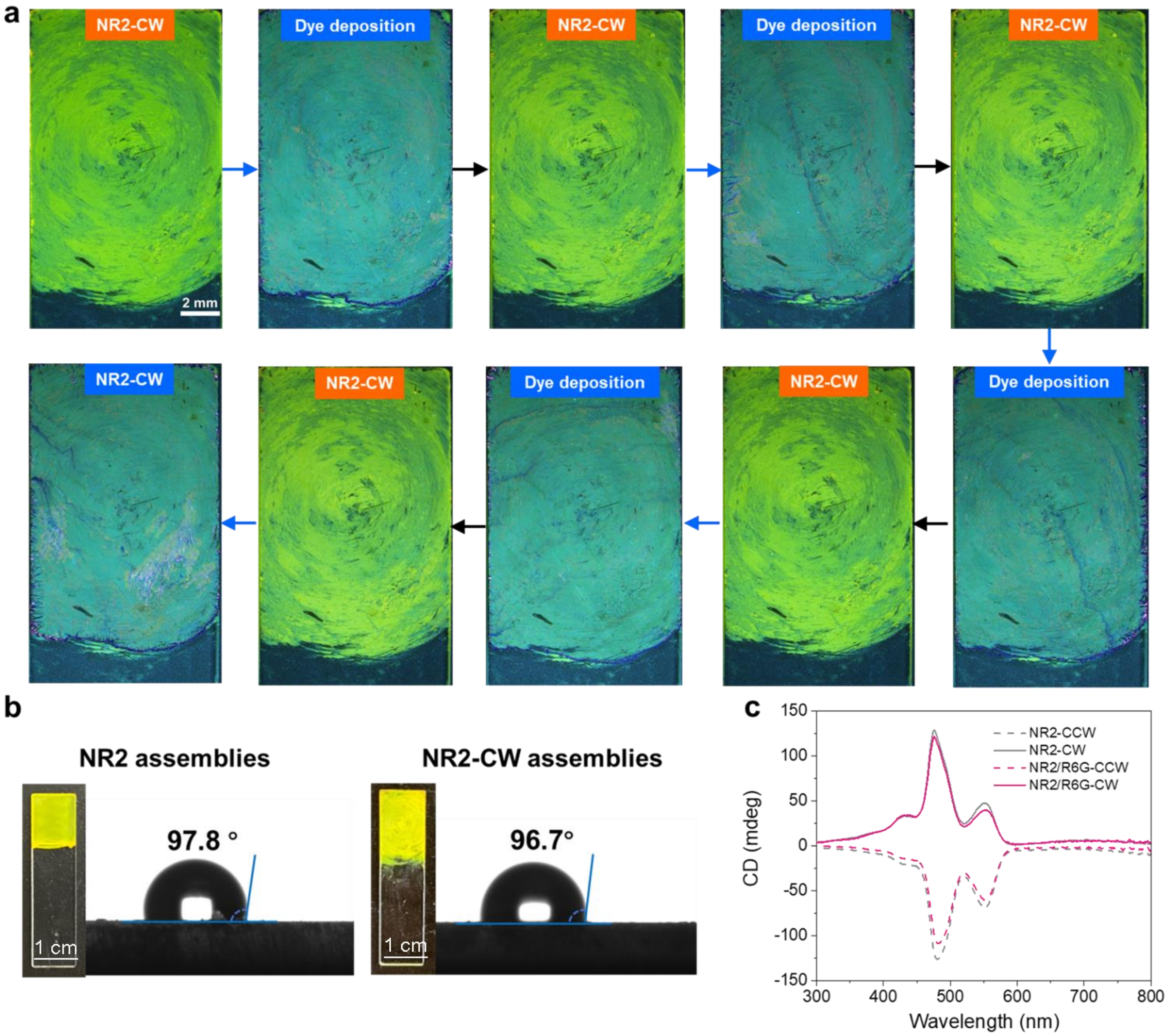

Supplementary Fig. 24. Relevant test related to wetting of non-polar dye molecules into pores. (a) Optical images of the NR2-CW assemblies with the deposition/removal of Sudan blue II dye, recoded under UV light. (b) Contact angle measurements of the NR2 assemblies and NR2-CW assemblies. (c) CD spectra of NR2/R6G-CW, NR2/R6G-CCW, NR2-CW, and NR2-CCW.

Supplementary Table 1. Synthetic conditions for various seeds for the NRs of distinct lengths and emission bands.

Type of seeds	PL of seeds (nm)	Addition of seeds (μL)	Type of CdSe-CdS NRs	Length of CdSe-CdS NRs (nm)	PL of CdSe-CdS NRs (nm)
Seed(519)	519	800	NR1	13.9±1.3	563
		450	NR2	22.5±1.6	564
		250	NR3	41.7±2.7	565
Seed(487)	487	450	NR2(540)	24.3±1.5	540
Seed(530)	530	450	NR2(571)	24.7±1.4	571
Seed(561)	561	450	NR2(601)	27.0±1.6	601

Supplementary Note 1. Inerparticle forces between CdSe/CdS NRs in self-assembled NR superlattices

First of all, concerning on the mechanism on the nanoscale chirality associated with the macroscale grinding, we believe that the rotational force from the macroscale grinding could break the spatial arrangement of NRs, rendering these NRs chiral. In fact, previous report showed that the handedness of helical supramolecular aggregates formed by achiral molecules can be well controlled by applying rotational forces during the self-assembly process[1,2]. To get insight on the chirality generation, we analyzed the pairwise interparticle energy between two NRs. The model of NRs was built with the diameter and length of 4 and 40 nm, respectively. The surface of NRs was coated with a layer of dodecanethiol with a ligand density of 4 ligands per nm^2 (Determined from ^{1}H-NMR method, data were not shown). In the self-assembled NR superlattices, the interaction energy between two neighboring NRs could be from the van der Waals attraction from the CdSe/CdS cores, van der Waals attraction from the aliphatic chains, and the elastic repulsion of the aliphatic chains. The van der Waals attractive energy from the CdSe/CdS cores is mainly determined by the Hamaker constant in hydrocarbon medium. Previous report indicates that the semiconductor core-core van der Waals interaction energy is low, comparable to the thermal energy k_BT (k_B, Boltzmann constant, T, absolute temperature)[3]. In fact, the interaction energy between two NRs of side-by-side packing is mainly from the intermolecular interactions of the surface aliphatic chains.

The interaction energy between aliphatic chains on two curved surfaces can be described by the optimal packing model (OPM) from Landman[4], which assumes that the ligands lying on the nanocrystal-nanocrystal line pack densely within a narrow volume, as a model to estimate the interaction contribution from the ligand shells. The OPM predicts the interparticle separation d to be

$$d = 2r[(1 + 3\frac{L}{r})^{1/3} - 1] \quad \text{(S1)}$$

Where r and L is the radius of the core particle and the length of aliphatic chains (~1.5 nm for dodecanethiol). For the NR of 2 nm in radius, an interparticle separation is calculated to be 1.9 nm, which agrees with our experimental observation from the TEM images. The van der Waals attraction (U_{C12}) between two nearest parallel alkyl chains of length L from N identical basic units ($L = N\lambda$) and separated by a distance D has been given by Salem[5]:

$$U_{C12} = \mathrm{A}\frac{3\pi}{8\lambda^2}\frac{L}{D^5} \quad \text{(S2)}$$

Where A is the Hamaker constant of methylene units. With Salem's conclusion that the attractive energy is correlated with the length of the alkyl chain, and with the attractive energy for C12 is calculated to be -9.6 k_BT/molecule, we could relate the overlapping length and interaction strength:

$$U_{attr} \approx (-4.8k_BT) \times (2L - d) \quad \text{(S3)}$$

The elastic repulsion energy between two C12 chains can be calculated on the basis of the elastic modulus (E), which is on the order of ~0.86 GPa for dodecanethiols[6]. Hence the elastic repulsion energy can be estimated to be

$$U_{el} \approx \frac{1}{2} \times \frac{EA_0}{L} \approx (17.2k_BT) \times (2L-d)^2 \quad \text{(S4)}$$

Where A_0 is the cross-sectional area of an alkyl chain ($A_0 \approx 0.25\, nm^2$). Considering the attractive and elastic repulsive energy between two parallel C12 chains, we claim that the overall contribution from the ligand-ligand interactions can be $\sim -1\ k_BT$ (The minus indicates the attractive energy). Considering the fact that two NRs are packed parallelly, the interactions from ligands can be described in formula:

$$U_{ligand} \approx N_{lig}[(-4.8k_BT) \times (2L-d) + (17.2k_BT) \times (2L-d)^2] \quad \text{(S5)}$$

Where N_{lig} is the total number of interacted ligands between two NRs. Since the OPM is built on the assumption that the ligands pack densely only with a narrow volume along the contact axis between two nearest neighbors, the N_{lig} depends on the length of NR and the curvature of NR. Concerning on the high curvature of the NR κ = 0.25 nm^{-1}, we assume that one pair of ligand-ligand interaction is possible at a unit length (~0.5 nm). Therefore, for the two NRs of side-by-side packing, their attractive energy from the surface ligands is estimated to be $\sim -80k_BT$ (~3.2×10^{-19} J).

The pressure we loaded on the NR assemblies ranges from 100 to 300 kPa (from 0.1 pN nm^{-2} to 0.3 pN nm^{-2}). For the surface area of the NR is approximately from tens to a few hundred of nm^2, we estimate that the applied force to each NR is a few tens of pN, which is capable of moving/rotating nanoscale objects. The work is on the order of 10^{-19} J, which is comparable to the attractive energy between two NRs.

Supplementary Note 2. The contribution of LD to the observed CD (CD_{OBS})

We analyzed the contribution of LD to the observed CD (CD_{OBS}) as follows:

$$CD_{OBS} = CD + LD\cos 2\beta sin\kappa + \frac{1}{6}\left[CDLBLD - CDLB^2 + \left(\frac{1}{2}\ln 10\right)^2 (CD^3 + CDLD^2)\right] \quad \text{(S6)}$$

where CB and LB denote the circular and linear birefringences, respectively. The term in square brackets can be ignored because of its negligibly small contribution, and the $\cos 2\beta sin\kappa$ term is nearly equal to 0.02 for a commercial CD spectropolarimeter equipped with a photoelastic modulator (PEM)[7]. Hence, the formula can be simplified as follows:

$$CD_{OBS} = CD + LD \times 0.02 \quad \text{(S7)}$$

Therefore, the contribution of LD in the CD_{OBS} in terms of percentage ($2LD/CD_{OBS}$%) is ~1.5% for NR2 assemblies subjecting to the CW grinding (Supplementary Fig. 6). In sharp contrast, the contribution from the LD effect is markedly greater in the NR2 assemblies experiencing the rubbing process in a linear fashion (Supplementary Fig. 5), and the value is estimated to be over 60%.

Supplementary Note 3. Comment on the film thickness that impacts the chiral nanoassemblies

To understand how the film thickness impacts the chiral nanoassemblies, we prepared the samples differing by the amounts of the NR2, which were subjected to the subsequent grinding treatments. After the deposition of the NR2 onto the substrate, the thickness of the film was determined by AFM measurements. Specifically, the as-prepared NR2 film was subjected to the scratching using a tweezer tip, which enables to determine the thickness of the film. Three distinct thicknesses of the NR2 films of ~300, ~540, and ~1100 nm, were obtained under the mass of NR2 of 0.2, 0.4, and 0.8 mg, respectively. After the grinding treatments under identical conditions, distinct CD intensities could be observed for these samples. The intensity of CD signal is markedly greater with the increase of the thickness of the film. When the sample is about 300 nm, rather low intensity of CD signal could be observed. This can also be understood by the *g*-factor at 490 nm. For the NR2 assemblies of 1100 nm in thickness, the *g*-factor could reach 0.01. In sharp contrast, it decreases to ~0.001 for the NR2 assemblies of 300 nm.

Supplementary Note 4. Comment on the correlation between the CD intensities and the distance (*d*) from the rotating center

To further confirm the correlation between the CD intensities and the distance (*d*) from the rotating center, we have performed multiple CD/CPL experiments at $d = 0$ mm, $d = 2.5$ mm, and $d = 5$ mm, referred as region 1, 2, and 3, respectively. The results were shown in Supplementary Fig. 9 and 10**.** The results showed that both the CD spectra and CPL spectra are very similar at three distinct regions. These results were highly reproducible, as confirmed by five independent grinding experiments. The dimensionless *g*-factor at 490 nm and g_{lum} at 575 nm of the samples at various regions were ~0.009 and ~0.05, respectively. These results confirmed that there was no observed difference of the CPL and CD intensity regarding the different *d*. Theoretically, the degree of cross-stacking of the NRs should depend on the distance between the NRs' position and the rotating center for a monolayer system. Here, the thickness of the NR is over 1 μm, which is roughly a few tens of layers of the NRs. During the grinding process, the NRs between interlayers could "interlock" each other, which eventually leads to the "equilibrium" structure after 21 s under 1.0 rad s^{-1}.

Supplementary Note 5. Additional comments on the deposition of dyes onto the chiral substrate

Concerning on the position of the dye molecules, the optical images of the sample during the dye deposition-removal were recorded under UV light, which showed distinct color changes (Supplementary Fig. 24a). It is worth noting that these dyes were homogeneously deposited on the NR assemblies, ruling out that these dyes were only deposited as aggregates in cracks. In fact, these NRs assemblies were hydrophobic in nature, confirmed by the large contact angle of ~97° (Supplementary Fig. 24b). Deposition of less polar dye molecules (such as Sudan I and Sudan blue II) enables their interaction with NRs through solvophobic force, which consequently results in the contact between NRs and dyes at the molecular level. In sharp contrast, deposition of polar dye molecules, such as Rhodamine 6G (R6G), would lead to the phase segregation between NRs and R6G aggregate. As a result, no induced CD signals from R6G could be observed when they were deposited onto the NR2-CW or NR2-CCW substrate (Supplementary Fig. 24c).

Supplementary References

1. Micali, N. et al. Selection of supramolecular chirality by application of rotational and magnetic forces. *Nat. Chem*. **4**, 201-207 (2012).

2. Sun, J. et al. Control over the emerging chirality in supramolecular gels and solutions by chiral microvortices in milliseconds. *Nat. Commun.* **9**, 2599 (2018).

3. Chen, Z., Moore, J., Radtke, G., Sirringhaus, H. & O'Brien, S. Binary nanoparticle superlattices in the semiconductor−semiconductor system: CdTe and CdSe. *J. Am. Chem. Soc.* **129**, 15702-15709 (2007).

4. Landman, U. & Luedtke, W. D. Small is different: energetic, structural, thermal, and mechanical properties of passivated nanocluster assemblies. *Faraday Discussions* **125**, 1-22 (2004).

5. Salem, L. Attractive forces between long saturated chains at short distances. **37**, 2100-2113 (1962).

6. DelRio, F. W., Jaye, C., Fischer, D. A. & Cook, R. F. Elastic and adhesive properties of alkanethiol self-assembled monolayers on gold. *Appl. Phys. Lett.* **94**, 131909 (2009).

7. Gillgren, H. et al. Morphology and molecular conformation in thin films of poly-γ-methyl-l-glutamate at the air−water interface. *Langmuir* **18**, 462-469 (2002).
